# The Impact of Probiotics on Acne Vulgaris: A Meta-Analysis of Randomized Controlled Trials

**DOI:** 10.7759/cureus.97010

**Published:** 2025-11-16

**Authors:** Marwa Mohamed, Ahsan Ullah, Rosi Hassan, Maha Hamza, Israa Mohamed, Muhammed Salam

**Affiliations:** 1 General Internal Medicine, Mid Cheshire Hospitals NHS Foundation Trust, Crewe, GBR; 2 Nutrition, Texas State University, San Marcos, USA; 3 Surgery, Ibrahim Malik Teaching Hospital, Khartoum, SDN

**Keywords:** acne vulgaris, gut-skin axis, meta-analysis, probiotics, randomized controlled trials, skin microbiome

## Abstract

Acne vulgaris is a multifactorial inflammatory skin disorder influenced by hormonal activity, microbial imbalance, and immune dysregulation. While conventional treatments such as antibiotics and retinoids remain effective, their long-term use is often limited by side effects, resistance, and poor adherence. This meta-analysis evaluated the efficacy of probiotics as an adjunct or alternative therapy for acne management. Four randomized controlled trials involving 227 participants were analyzed, showing that probiotic supplementation reduced acne severity scores (OR 0.48; 95% CI 0.29-0.79) and non-inflammatory lesion counts (mean difference (MD) −4.62; 95% CI −8.10 to −1.15) compared with controls. A trend toward improvement in inflammatory lesions was observed (MD −2.03; 95% CI −5.46 to 1.41) but was not statistically significant. Heterogeneity across studies ranged from moderate to high, reflecting variation in probiotic strains, formulations, and treatment durations. While these findings suggest a potential benefit of probiotics, the limited number and quality of trials warrant cautious interpretation. Larger, standardized clinical studies are needed to confirm efficacy and identify optimal probiotic regimens for acne management.

## Introduction and background

Acne vulgaris is one of the most common skin diseases in the world, affecting people of different age groups; nevertheless, the majority of cases surface during adolescence [1]. It is a multifactorial ailment characterized by the presence of comedones, papules, pustules, and nodules due to follicular hyperkeratinization, excessive sebum production, colonization of *Cutibacterium acnes* (*C. acnes*), and inflammation [2]. Acne is self-limiting most of the time; however, it can cause an impact on self-esteem, mental health, and quality of life, thereby prompting continuous attempts toward finding effective treatment strategies [3]. Conventional treatments include benzoyl peroxide, retinoids, topical and systemic antibiotics, and hormonal therapy [4]. Concerns about antibiotic resistance, side effects of treatment, and patient compliance have, however, increased interest in other adjunctive or alternative therapies, such as probiotics.

The gut-skin axis has gathered significant attention in the field of dermatological research, delineating the bidirectional relationship between gastrointestinal health and skin conditions [5]. Early observation of this association goes back to the early 20th century, when dermatologists suggested that intestinal dysbiosis might play a role in some skin disorders [6]. Further understanding of this was augmented by studies that examined the alterations in gut microbiota of healthy and acne patients [7]. These responses were related to changes in systemic inflammation, immune regulation, and metabolic processes that have been involved in acne pathogenesis [8]. Defined as live microorganisms that confer health benefits when administered in an adequate amount [9], probiotics have been studied with the intention of restoring microbial balance and modulating immune responses, therefore making this an important theory for acne therapy [10].

Some of the initial studies on probiotics and acne were small-scale trials [11]. The early claims for probiotics, especially *Lactobacillus* and *Bifidobacterium*, were of improving gut health and reducing systemic inflammation, which leads to changes in skin conditions. Many early studies on probiotics involved supplementation of probiotics along with traditional treatment for acne and indicated greater effectiveness and fewer gastrointestinal side effects from antibiotics. However, these studies were not standardized [12].

Due to the vast interest in probiotics, clinical trials began to reveal the mechanisms by which probiotics may affect acne severity. Some of these trials have indicated the influence of probiotics on lesion counts and overall skin condition. The working hypothesis is that probiotics can modulate both systemic and local inflammation by enhancing the regulatory T-cell activity, reducing pro-inflammatory cytokines, and competing with *C. acnes* for adhesion sites [13]. Probiotics have also been hypothesized to modulate insulin sensitivity and hormone status, crucial factors in the pathogenesis of acne, particularly in those with insulin resistance or polycystic ovary syndrome (PCOS) [14]. Although positive in findings, the studies differ in terms of some major factors, including probiotic strains and dosages used, and study designs. 

Observational studies have further confirmed that probiotics can exert molecular mechanisms against acne, along with clinical trials. It has been shown that some probiotic strains can produce antimicrobial peptides that prevent the growth of *C. acnes*, while others improve skin barrier function by enhancing ceramide production [15]. Studies also suggest that probiotics modulate the gut microbiome and aid in reducing systemic lipopolysaccharide (LPS) levels, LPS being an agent that promotes systemic inflammation and acne exacerbation [16]. Another aspect of ongoing research is whether probiotics can help regulate oxidative stress, an inadequately assessed variable in acne pathophysiology, which further promotes their use as an adjunct treatment [17].

Despite growing evidence of the potential benefits of probiotics in acne management, skepticism persists due to inconsistent findings across studies. Variations in probiotic strains, study duration, participant demographics, and outcome measures have led to divergent conclusions, highlighting the need for systematic evaluation. While some trials demonstrate marked reductions in acne severity, others report only modest or negligible effects, raising questions about which patient populations may benefit most from probiotic therapy [18]. Given rising antibiotic resistance and the demand for safer, more sustainable therapies, an evidence-based assessment of probiotics in acne treatment is warranted.

This meta-analysis aims to systematically evaluate the efficacy of probiotics in acne vulgaris by synthesizing available clinical data and addressing the heterogeneity among studies. Specifically, it examines probiotic effects on inflammatory and non-inflammatory lesions, as well as overall acne severity, to clarify their therapeutic role and guide future research toward standardized, evidence-based probiotic interventions in dermatology.

## Review

Methods

Search Strategy

This meta-analysis was conducted in accordance with the PRISMA (Preferred Reporting Items for Systematic Reviews and Meta-Analyses) guidelines. A systematic literature search was performed on March 28th, 2025, using databases including PubMed, Cochrane Central Register of Controlled Trials (CENTRAL), and ClinicalTrials.gov. No restrictions were applied in terms of publication date. The search strategy utilized a combination of MeSH terms and keywords related to “probiotics,” “acne vulgaris,” “cutaneous microbiota,” and “gut-skin axis.” The full, replicable search strings for each database are provided in Appendix 1. No language or time filters were initially applied, but only English-language articles were retained for inclusion.

*Eligibility Criteria* 

Eligible studies included randomized controlled trials (RCTs) that investigated the use of probiotics in the treatment of acne vulgaris in human participants aged over 12 years. Studies had to compare probiotics either as monotherapy or adjunct therapy versus placebo or standard acne treatments. Studies were excluded if they were observational studies, case reports, editorials, reviews, commentaries, letters, animal or in vitro studies, or lacked extractable data on acne outcomes (e.g., lesion count, severity score). Non-English articles were also excluded from the analysis.

*Study Selection* 

Two authors (M.M. and A.U.) independently screened all retrieved articles by title, abstract, and subsequently full text, applying the inclusion and exclusion criteria. Studies were considered eligible if they (1) included patients with a clinical diagnosis of acne vulgaris, (2) compared probiotic intervention with control or placebo, and (3) reported at least one clinical outcome, such as total lesion count, inflammatory and non-inflammatory lesions, or acne severity index (ASI). Any discrepancies during the selection process were resolved through discussion with a third reviewer (M.H.).

*Data Extraction and Quality Assessment* 

A standardized data extraction form was used to collect study characteristics and relevant outcome data, including the first author, year of publication, sample size, participant demographics, type and duration of probiotic intervention, comparator treatment, and reported outcomes. The quality of RCTs was assessed using the Cochrane Risk of Bias tool.

Outcomes and Statistical Analysis 

Primary outcomes assessed included changes in total acne lesion count and severity scores. Secondary outcomes included changes in inflammatory lesions, non-inflammatory lesions, and reported side effects. For dichotomous outcomes, odds ratios (ORs) with 95% confidence intervals (CIs) were calculated. As all continuous outcomes (inflammatory and non-inflammatory lesion counts) were measured on the same scale (i.e., 'lesion count'), the mean difference (MD) was the appropriate measure, and the use of standardized mean differences (SMDs) was not necessary. MDs with 95% CIs were pooled. The Mantel-Haenszel method was used for dichotomous variables and the inverse variance method for continuous variables. Heterogeneity among studies was assessed using the I² statistic, with values above 50% indicating substantial heterogeneity. A random-effects model was applied in cases of significant heterogeneity. A P-value <0.05 was considered statistically significant. Statistical analysis was conducted using Review Manager (RevMan) version 5.4 (The Cochrane Collaboration, Copenhagen, Denmark). Formal sensitivity or subgroup analyses were not feasible given the limited number of included trials (n=4) and the high statistical heterogeneity.

**Figure 1:**
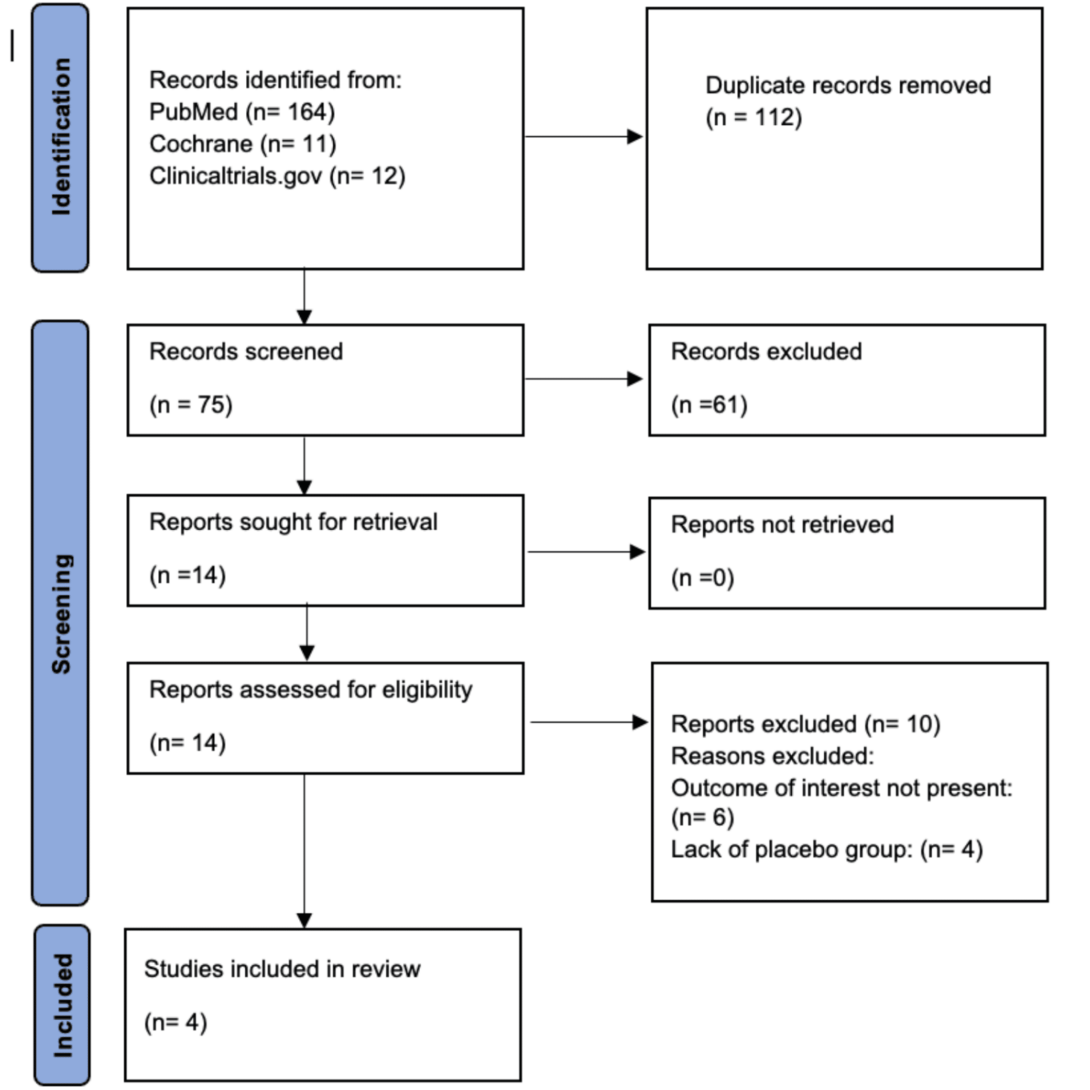
PRISMA flowchart PRISMA, Preferred Reporting Items for Systematic Reviews and Meta-Analyses.

As shown in the PRISMA flowchart (Figure 1), four randomized controlled trials (RCTs) were isolated for analysis. Table 1 summarizes the characteristics of the four RCTs included in this study, which assess the role of probiotics in acne management. The studies were conducted in various countries, including Spain, Korea, and Italy. Sample sizes varied across studies, ranging from 14 to 40 participants in each group. Participant demographics such as age, gender distribution, and body mass index (BMI) were reported to different extents, with some studies lacking specific data. Probiotic interventions were delivered either orally in capsule form or topically as a lotion. The composition of the probiotics differed among studies, including strains such as *Lacticaseibacillus rhamnosus*, *Bifidobacterium breve*, *Lactobacillus plantarum*, and *Enterococcus faecalis*. This variation in formulations, delivery routes, and participant profiles reflects methodological heterogeneity, which should be considered when comparing the efficacy outcomes across the included studies.

**Table 1:** Study characteristics table RCT, randomized controlled trial.

Study	Country	Study Design	Sample Size (Placebo/Probiotic)	Age (Placebo/Probiotic)	BMI (Placebo/Probiotic)	Male Gender (Placebo/Probiotic)	Probiotic Form	Probiotic Composition
Kang et al. 2009 [19]	Korea	RCT	33/37	Not mentioned	Not mentioned	Not mentioned	Lotion (topical)	Enterococcus faecalis
Rinaldi et al. 2022 [20]	Italy	RCT	28/27	23.70 ± 7.59/23.43 ± 7.01	Not mentioned	16/13	Capsule (oral)	*Bifidobacterium breve*, *Lacticaseibacillus casei*, *Ligilactobacillus salivarius*, *Lactobacillus acidophilus*
Eguren et al. 2024 [21]	Spain	RCT	34/40	18.03 ± 0.87/20.13 ± 0.80	21.84 ± 0.73/22.68 ± 0.98	15/10	Capsule (oral)	*Lacticaseibacillus rhamnosus* (CECT 30031) and *Bifidobacterium lactis* (CECT 8145)
Kim et al. 2021 [22]	Korea	RCT	14/14	23.86 ± 0.80/24.29 ± 0.73	21.39 ± 0.55/20.74 ± 0.64	7/5	Capsule (oral)	*Lactiplantibacillus plantarum* CJLP55

**Figure 2:**
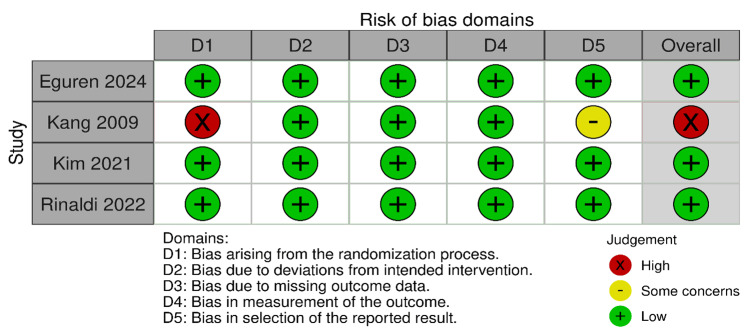
Cochrane Risk of Bias tool Refs. [19-22].

Risk of bias was assessed using the Cochrane ROB2 tool and is elaborated on in Figure 2. The risk of bias for the four included RCTs was assessed using the Cochrane Risk of Bias tool. The findings revealed that three of the four studies [20-22] demonstrated a low overall risk of bias, with low risk judgments across all five domains. One study, Kang 2009 [19], was assessed as having a high overall risk of bias. This was primarily driven by a high risk of bias in the 'randomization process' (D1) and 'some concerns' regarding 'bias in selection of the reported result' (D5).

The outcomes assessed in this meta-analysis included persistent acne based on acne severity scores, non-inflammatory lesion count, and inflammatory lesion count. The acne severity was assessed using the Investigator’s Global Assessment (IGA) or the Global Acne Grading System (GAGS), ASI, and total lesion count [1-4].

**Figure 3:**
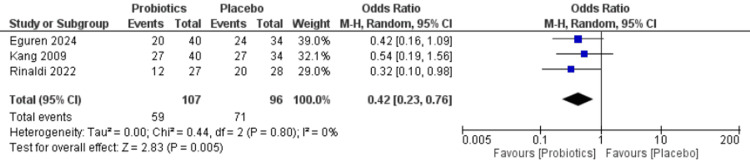
Forest plot for persistent acne (severity index) Refs. [19-21].

Persistent acne (based on acne severity scores): The forest plot in Figure 3 examines the odds of persistent acne severity in probiotics versus placebo groups. The results indicate a significant reduction in the likelihood of persistent acne with the use of probiotics. The findings are consistent across studies, with no statistical heterogeneity (I² = 0%, P = 0.80), reinforcing the potential of probiotics in mitigating acne severity. 

**Figure 4:**
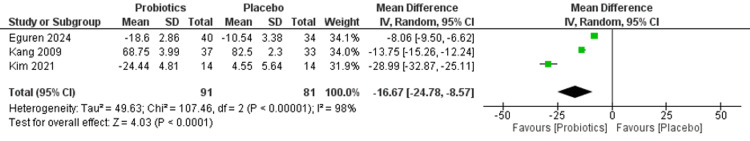
Forest plot for non-inflammatory lesion count Refs. [19,21,22].

Non-inflammatory lesion count: The forest plot in Figure 4 evaluates the MD in non-inflammatory lesion count between probiotics and placebo groups. The analysis favors probiotics, showing a significant reduction in non-inflammatory lesions. However, there was substantial statistical heterogeneity, I² = 98% (P < 0.00001), suggesting differences in study populations or methodologies. Despite this, the overall effect remains statistically significant, supporting the beneficial role of probiotics in reducing non-inflammatory acne lesions.

**Figure 5:**
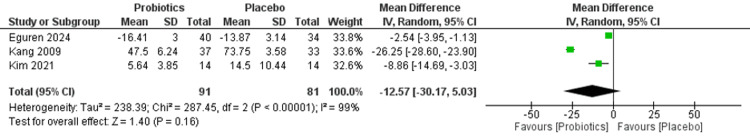
Forest plot for impact on inflammatory lesions Refs. [19,21,22].

Inflammatory lesions: The forest plot in Figure 5 assesses the MD in inflammatory lesion count between probiotics and placebo groups. While the analysis suggests a potential reduction in inflammatory lesions, the CI includes no effect, making the result statistically inconclusive. Additionally, substantial statistical heterogeneity exists among studies, I² = 99% (P < 0.00001), indicating differing responses to probiotics. This suggests that while probiotics may have a beneficial trend, further research is necessary to confirm their impact on inflammatory acne lesions.

Discussion

This meta-analysis provides a comprehensive evaluation of the potential therapeutic role of probiotics in managing acne vulgaris, synthesizing data from four RCTs. The results suggest that probiotic supplementation, either as monotherapy or adjunct therapy, may have a favorable impact on acne severity, especially for non-inflammatory lesions. While these findings are encouraging, the strengths and limitations of the current evidence must be highlighted for a more comprehensive and nuanced approach. 

The observed reduction in acne severity scores in the probiotic groups compared to placebo suggests a potential role for probiotics in moderating the chronic and inflammatory components of acne. However, this finding must be interpreted with caution, as the strength of this evidence is limited. The assertion that this effect is consistent across trials must be discussed more critically in light of the impact of study heterogeneity and small trial sizes, which restrict the generalizability of these findings. The mechanisms likely contributing to these outcomes include immunomodulation, suppression of systemic and cutaneous inflammation, and competitive inhibition of *C. acnes* colonization by beneficial microorganisms. Certain strains, such as *Lacticaseibacillus rhamnosus* and *Bifidobacterium breve*, which were commonly employed in the studies included, have been shown in preclinical and clinical models to potentially enhance skin barrier integrity and reduce pro-inflammatory cytokine production, both of which are pivotal in acne pathogenesis.

The reduction in non-inflammatory lesion counts further supports the hypothesis that probiotics can affect earlier stages of acne development, such as comedogenesis. This is significant because non-inflammatory lesions typically precede inflammatory flares and represent a window for early intervention. The consistency in these results despite methodological heterogeneity could strengthen the biological plausibility of probiotics' effects. Nevertheless, high heterogeneity levels suggest that factors such as probiotic formulation, dosage, treatment duration, and delivery method likely influence treatment response. For instance, the topical use of *Enterococcus faecalis* in the Kang et al. [19] study contrasts with the oral administration of multi-strain probiotics in the Rinaldi et al. [20] and Eguren et al. [21] studies, potentially affecting the route-dependent outcomes on cutaneous versus systemic inflammation. 

The impact of probiotics on inflammatory lesions was less conclusive. Although the data trends in favor of probiotics, the CIs overlapped with the null effect, and heterogeneity among studies was substantial [22]. This may be attributed to variations in study populations, such as differences in baseline acne severity, hormonal status, or presence of metabolic comorbidities like insulin resistance or PCOS [23]. These underlying factors could mediate differential responses to probiotic therapy, particularly in the context of inflammation. Additionally, some probiotics may exert a more prominent effect on gut inflammation and systemic immune markers, which might not translate directly to visible reductions in inflammatory lesions within the relatively short durations of these trials (often under 12 weeks).

Another important limitation is the lack of standardization across probiotic strains and formulations. Different probiotic species have varying properties and mechanisms of action, and few trials have undertaken comparative evaluations of specific strains. This variation poses a challenge in recommending a "universal" probiotic therapy for acne. Moreover, most studies did not report on participants’ baseline gut microbiome profiles or dietary habits [24], which are critical modulators of probiotic efficacy. The inter-individual variability in gut flora may explain the inconsistent outcomes and limit the generalizability of these findings across populations.

The sample sizes in all four trials were relatively small, ranging from 14 to 40 participants per arm. While the results achieved statistical significance in several outcomes, small sample sizes reduce the power to detect subtle effects and increase the likelihood of type II errors. Larger, multicenter RCTs with robust methodologies are needed to confirm these preliminary findings. Furthermore, the follow-up periods in included studies were generally short-term, which may not reflect long-term efficacy or safety of probiotic use. Since acne is a chronic and often relapsing condition, longer studies are essential to determine whether probiotics can offer sustained benefits or prevent recurrence.

Safety and side-effect profiles were not uniformly reported across studies, although probiotics are generally regarded as safe for the general population [25]. Importantly, their favorable tolerability profile compared to antibiotics or retinoids may make them especially appealing to individuals seeking gentler, natural treatments [26]. The potential to reduce gastrointestinal side effects when used in conjunction with antibiotics also opens avenues for incorporating probiotics into multi-modal acne treatment regimens. 

Future research should be designed to address the critical limitations identified in this review. Given the significant impact of study heterogeneity and small trial sizes, it remains unknown whether specific subgroups, such as adolescents versus adults, males versus females, or patients with insulin resistance, derive more pronounced benefit from probiotic intervention. An intriguing area for future trials would be to explore personalized probiotic therapies. Tailoring regimens based on an individual’s microbiota profile or inflammatory markers may enhance therapeutic outcomes, but this is speculative and would require robust, large-scale studies to investigate.

## Conclusions

This meta-analysis suggests a potential role for probiotics in reducing acne severity, particularly non-inflammatory lesions. The included RCTs indicate that probiotics, as oral or topical formulations, may serve as a promising adjunctive therapy with minimal adverse effects. However, the current evidence suggests a potential rather than definitive benefit. This is limited by notable heterogeneity in probiotic strains, delivery methods, and study designs, which restricts the generalizability of findings. While results are encouraging, especially considering rising antibiotic resistance, further large-scale, standardized clinical trials are needed to confirm efficacy, identify optimal probiotic formulations, and define clear therapeutic regimens.

## Appendices

Full Search Strategies

1. PubMed Database Search Strategy

((("Probiotics" OR "probiotics") OR ("Microbiota" OR "Gastrointestinal Microbiome" OR "Skin / microbiology" OR "cutaneous microbiota" OR "skin microbiota" OR "gut-skin axis")) AND (("Acne Vulgaris" OR "acne vulgaris" OR "acne"))) AND ("randomized controlled trial" OR "randomized" OR "placebo")

2. Cochrane Central Register of Controlled Trials (CENTRAL) Search Strategy

([probiotics] OR [microbiota] OR [gut-skin axis] OR [cutaneous microbiota]) AND ([acne] OR [acne vulgaris])

3. ClinicalTrials.gov Search Strategy

(probiotics OR microbiota OR "gut-skin axis") AND ("acne vulgaris" OR acne)
